# Treatment response and preliminary efficacy of hepatic tumour laser ablation under the guidance of percutaneous and endoscopic ultrasonography

**DOI:** 10.1186/s12957-019-1677-6

**Published:** 2019-08-05

**Authors:** Weilu Chai, Qiyu Zhao, Huiming Song, Chao Cheng, Guo Tian, Tian’an Jiang

**Affiliations:** 1 Department of Ultrasonography, The First Affiliated Hospital, College of Medicine, Zhejiang University, #79 Qingchun Road, Hangzhou, 310003 Zhejiang Province People’s Republic of China; 2 Department of Hepatobiliary and Pancreatic Surgery, The First Affiliated Hospital, College of Medicine, Zhejiang University, #79 Qingchun Road, Hangzhou, 310003 Zhejiang Province People’s Republic of China; 3 Collaborative Innovation Center for Diagnosis and Treatment of Infectious Diseases, The First Affiliated Hospital, College of Medicine, Zhejiang University, #79 Qingchun Road, Hangzhou, 310003 Zhejiang Province People’s Republic of China

**Keywords:** Laser ablation, Endoscopic ultrasonography, Liver cancer, Interventional ultrasonography

## Abstract

**Background:**

Studies focusing on the treatment effectiveness of endoscopic ultrasonography (EUS)-guided laser ablation (LA) for hepatic tumours or the technical differences from percutaneous ultrasonography (US)-guided LA are lacking. The objective of this study was to evaluate the treatment response and preliminary efficacy of US- and EUS-guided LA for hepatic tumours.

**Methods:**

We performed a prospective analysis of treatment response and preliminary efficacy in 92 consecutive patients who underwent US-LA and EUS-LA from January 2015 to June 2017. The primary endpoint was complete tumour ablation (CTA). The secondary endpoint was 12-month local tumour progression (LTP).

**Results:**

Among a total of 120 hepatic lesions, 20 lesions were ablated under the guidance of EUS. The application of the multi-fibre technique (0, 0% vs. 69, 69.0%, *p* < 0.01), tumours located in the left lobe (18, 90.0% vs. 28, 28.0%, *p* < 0.01) and multi-session ablation (4, 20.0% vs. 4, 4.0%, *p* = 0.009) were factors found to be significantly different between the EUS-LA and US-LA groups. The CTA was achieved in 94% of patients in the US-LA group and 100% of patients in the EUS-LA group (*p* = 0.261). Twelve-month LTP was observed in 8.5% of the patients in the US-LA group and 15.0% of the patients in the EUS-LA group with no significant difference between the two groups (*p* = 0.372). The multivariate analysis identified that the tumour diagnosis (*p* = 0.004; 95% CI, 0.039–0.547) was the only independent risk factor associated with 12-month LTP.

**Conclusions:**

Patients in the EUS-LA and US-LA groups shared as similar treatment response and preliminary efficacy in the treatment of hepatic tumours. A hybrid LA approach to nonsurgical hepatic tumours appeared to be reasonable.

## Background

Over the last two decades, radiofrequency ablation (RFA) has been recommended as a curative option for early hepatocellular carcinoma (HCC), and it represents the most common ablative technique that affords better local tumour control and long-term clinical outcomes. Percutaneous ultrasonography (US)-guided laser ablation (LA) has been reported to be a minimally invasive therapy and extremely well-tolerated without severe complications. The ideal candidates for LA are those with well-differentiated histology, non-infiltrating growth, naive tumours, a first treatment session and normal bilirubin levels [1, 2]. Previous studies demonstrated that LA retained an equal ablation efficacy to that of RFA, and the published complete response rate ranged from 82% to 97% [3, 4].

In the past 30 years, endoscopic ultrasonography (EUS) has been widely used in clinical practice. It gained popularity with the use of EUS for the diagnosis and staging of malignancies and has particularly increased the diagnostic rate for pancreaticoduodenal lesions.

When certain hepatic tumour are in the close proximity to gastrointestinal structures, the visualization via EUS is more direct and distinct than percutaneous US [5, 6]. To date, the introduction of curvilinear EUS has made possible the booming development of EUS-guided fine-needle aspiration (FNA). Subsequently, EUS-guided fine-needle injection (FNI), especially the EUS-guided ethanol injection, emerged as a new method for the chemo-ablation of pancreatic cysts or hepatic tumours. Guided by a needle puncture, the Neodymium: Yttrium Aluminium Garnet (Nd:YAG) laser fibre is inserted at different angles and allows multiple punctures. It has been reported that EUS-guided LA remains a feasible alternative in the situations where the target is hard to reach or where there is a lack of an appropriate puncture route from the percutaneous approach [5–13].

However, studies focusing on the treatment effectiveness of EUS-guided LA for hepatic tumours or the technical differences from percutaneous US-guided LA are lacking. The purpose of our analysis was to evaluate the treatment response and preliminary efficacy of percutaneous US- and EUS-guided LA for hepatic tumours.

## Methods

### Study population

A total of 92 consecutive patients who underwent either percutaneous US- or EUS-guided LA at our hospital from January 2015 to June 2017 were prospectively enrolled and the study was registered in Clinicaltrials.gov ID: NCT02816944. All procedures performed in studies involving human participants were in accordance with the ethical standards of the institutional research committee and with the 1964 Helsinki declaration and its later amendments or comparable ethical standards. Percutaneous US- and EUS-guided LA to solid tumour was approved by the institutional review board of our hospital, and the written informed consent from each participant was obtained in our study. The baseline demographic and clinical characteristics of the included patients were collected from electronic medical records. The diagnosis of all the included patients was confirmed by pathologic examination or non-invasive criteria that was divided into hepatocellular carcinoma (HCC) and non-HCC nodules (hepatic metastasis or intrahepatic cholangiocarcinoma) [14]. The size, number and location of the target lesion was previously evaluated by at least two imaging modalities. When the target lesion was in close proximity (< 5 mm) to vital structures (stomach/bowel loops/heart/gall bladder/hepatic hilum/Glisson’s capsule), it was considered to be located at a high-risk position.

The inclusion criteria were as follows: (1) poor surgical candidates or not eligible for surgery, (2) single tumour ≦ 3 cm in diameter and the number of visible tumours were no more than 3 and (3) hepatic tumours were visualized by percutaneous US or EUS with the assistance of contrast medium.

Exclusion criteria were as follows: (1) age > 80 years; (2) radiological evidence of uncontrolled extra-hepatic carcinoma; (3) portal vein thrombosis or hepatic vein thrombosis; (4) uncontrolled liver disease decompensation (gastrointestinal bleeding, encephalopathy, refractory ascites, bacterial infection); (5) severe clotting impairment (platelet count less than 38 × 10^9^/L or prothrombin time with international normalized ratio greater than the normal value); (6) Child-Pugh class C; (7) multiple organ failure; (8) suspicious gastrointestinal perforation; (9) intravenous anaesthesia contraindication; (10) thoraco-abdominal aortic aneurysm; and (11) acute intra-cerebral haemorrhage [15].

### Laser ablation

All the procedures of either percutaneous US- or EUS-guided LA were performed in patients under conscious sedation by two operators in the same team who had at least 10 years of experience in thermal ablation. The guiding method of LA (US or EUS) was previously decided by the operator in our study. If the tumour was poorly visualized in US imaging or was hardly reached by percutaneous approach, especially the one adjacent to gastric wall or in the possible range of EUS scanning, EUS-guided LA was tried and performed. EUS was performed using a curvilinear echoendoscope (GF-UCT240, Olympus Medical Systems, Tokyo, Japan) to visualize the hepatic tumours through the gastric wall. The real-time US system (MyLab90 XVision, ultrasound system, Esaote, Florence, Italy) with an ultrasound transducer (3.5-MHz convex array probe) was used to navigate percutaneous tumour visualization and puncture.

Nd:YAG lasers, with a wavelength of 1064 nm, were commercially available (Echolaser, Esaote, Florence, Italy) and consisted of an ultrasound device and a diode laser unit. In the percutaneous approach, which was guided by US, the 1.5-m bare fibre (300 μm, quartz-core) with a flat tip was inserted through 22-gauge needle into the end of the sheath and carefully advanced out of the needle tip (at least 1.0-cm bare tip) to propagate the therapeutic beams [16]. The needle was placed at the frontal edge of the tumour prior to starting the treatment. The number, length and arrangement of needles of the fibres were determined in accordance with the size, shape and location of the tumour. In the trans-gastric method, the 3.0-m bare fibres (300 μm, quartz-core) was used and the single-fibre technique with a multi-puncture LA was adopted.

Once the fibres were accurately positioned, the laser was turned on at a power of 5.0 W, and the required energy was delivered (1800 J per fibre) for a single session of treatment, resulting in tumour coagulation. The one-fibre ablative zone was 1.2–1.5 cm in diameter. It was possible to achieve a sufficient necrotic margin by the strategy of multi-fibre LA or multi-session LA. Contrast-enhanced ultrasonography (CEUS), performed during or immediately after the PLA procedure by SonoVue (contrast agent, Bracco, Italy), is an important method for evaluating whether the ablation margin is sufficient. If thermo-induced gases interfered with the intraprocedure CEUS, reassessment after 20 min or 1 day was suggested to be more accurate than immediate assessment. The LA procedure data, such as the number of fibres, number of session, the total delivered energy and the monitoring method, were recorded after the procedure.

### Complication and assessment of LA efficacy

The primary endpoint was complete tumour ablation (CTA), defined as the absence of any contrast uptake within or at the periphery of the ablative zone. One month after either one session or multiple LA sessions, CTA was confirmed by contrast-enhanced computed tomography (CT) or magnetic resonance imaging (MRI). Complications and side effect of LA were recorded according to the Society of Interventional Radiology guidelines. The requirement of blood transfusion or interventional treatment was considered a major complication; other conditions were considered minor complications [3, 4]. A follow-up evaluation was performed at 12 months via an outpatient interview, from which the secondary endpoint of local tumour progression (LTP) was identified and recorded. All the imaging results were obtained in consensus with at least two radiologists. LTP was defined as the progression of a newly enhanced area on contrast-enhanced CT or MRI contiguous with (< 2 cm) or within the primary ablative zone.

### Statistical analysis

Continuous variables are expressed as the means ± SD and compared using Student’s *t* test or nonparametric test (in the non-normal distributional data or the data with heterogeneity of variance). Categorical variables are presented as percentages and compared using the chi-square or the Fisher’s exact test, as appropriate. The rates of complications, CTA and LTP between the US-LA and EUS-LA groups were compared using the chi-square or the Fisher’s exact test. The risk factors for 12-month LTP were analyzed in a forward multivariable logistic-regression model that selected ‘12-month LTP’ as the positive event. A *p* value of < 0.05 was considered statistically significant. All statistical analyses were performed using SPSS (version 20.0).

## Results

### Study population

A total of 92 patients who underwent either percutaneous US- or EUS-guided LA at our hospital from January 2015 to June 2017 was prospectively included. Table 1 summarizes the lesion characteristics in the EUS-LA and US-LA groups. The mean age of the included 92 patients was 59.58 ± 11.71 years, 99.0% of the patients were Child-Pugh class A, 77.2% of the patients were male and 67.4% of patients had liver cirrhosis. The hepatic tumours were divided into HCC (89, 74.1%), intrahepatic cholangiocarcinoma (ICC) (11, 9.2%) and hepatic metastasis (20, 16.7%). A total of 70 tumours were determined to be located at a high-risk position: 35 tumours were strictly adjacent to the major vessels, such as the first- or second-grade portal vein, hepatic vein or inferior vena cava (IVC); 10 tumours, adjacent to the hepatic hilum (within 5 mm); and 25 tumours had a margin ≦ 5 mm from some vital structures, such as the gallbladder, kidney, heart and gastrointestinal tract.

**Table 1:** Lesion characteristics in the EUS-LA and US-LA groups

	US-LA (*n* = 100)	EUS-LA (*n* = 20)	*p* value
History of thermal ablation/Y	67 (67.0%)	16 (80.0%)	0.250
Multiple hepatic tumours/Y	64 (64.0%)	9 (45.0%)	0.112
Tumour diagnosis/HCC	77 (77.0%)	12 (60.0%)	0.113
Poorly differentiated tumour/Y	22 (22.0%)	3 (15.0%)	0.482
Recurrent lesion/Y	88 (88.0%)	20 (100%)	0.102
High-risk position/Y	56 (56.0%)	14 (70.0%)	0.246
Tumour position/left lobe	28 (28.0%)	18 (90.0%)	< 0.01
Maximum tumour diameter/mean ± SD, cm	1.72 ± 0.58	1.79 ± 0.75	0.676
Multi-fibre technique/Y	69 (69.0%)	0 (0%)	< 0.01
Multi-session ablation/Y	4 (4.0%)	4 (20.0%)	0.009
Delivered energy/mean ± SD, J	2927.94 ± 1690.50	2449.00 ± 1579.08	0.245

*US* ultrasonography, *EUS* endoscopic ultrasonography, *LA* laser ablation, *SD* standard deviation, *Hb* haemoglobin, *AFP* alpha-fetoprotein, *HCC* hepatocellular carcinoma, *CTA* complete tumour ablation, *LTP* local tumour progression, *Y* yes

### EUS-LA and US-LA

**Fig. 1:**
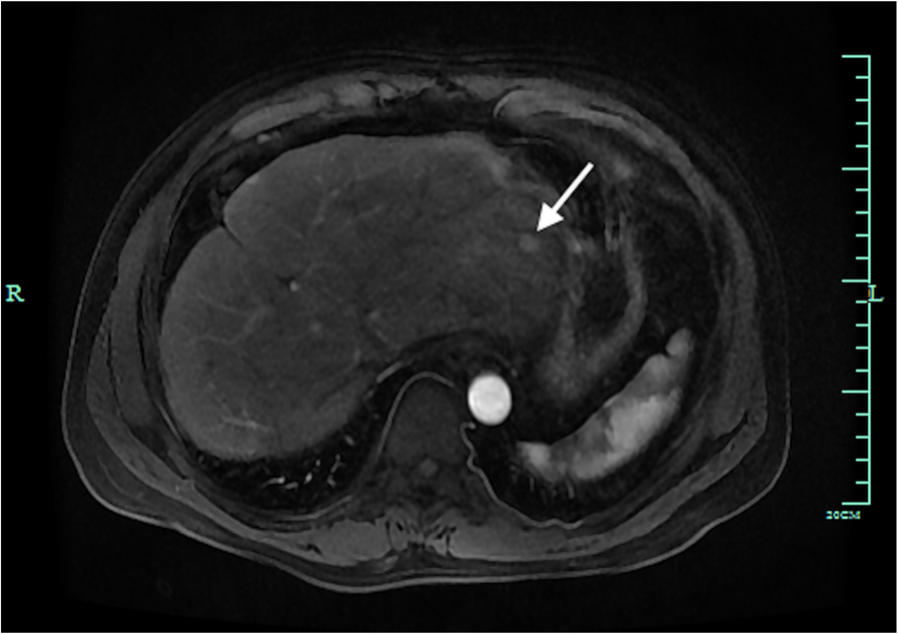
EUS-guided LA for HCC in the left external lobe of the liver is shown. In the arterial phase of the MRI, a hypo-intensity nodule located in the external lobe of the liver is hyper-enhanced (arrowhead)

**Fig. 2:**
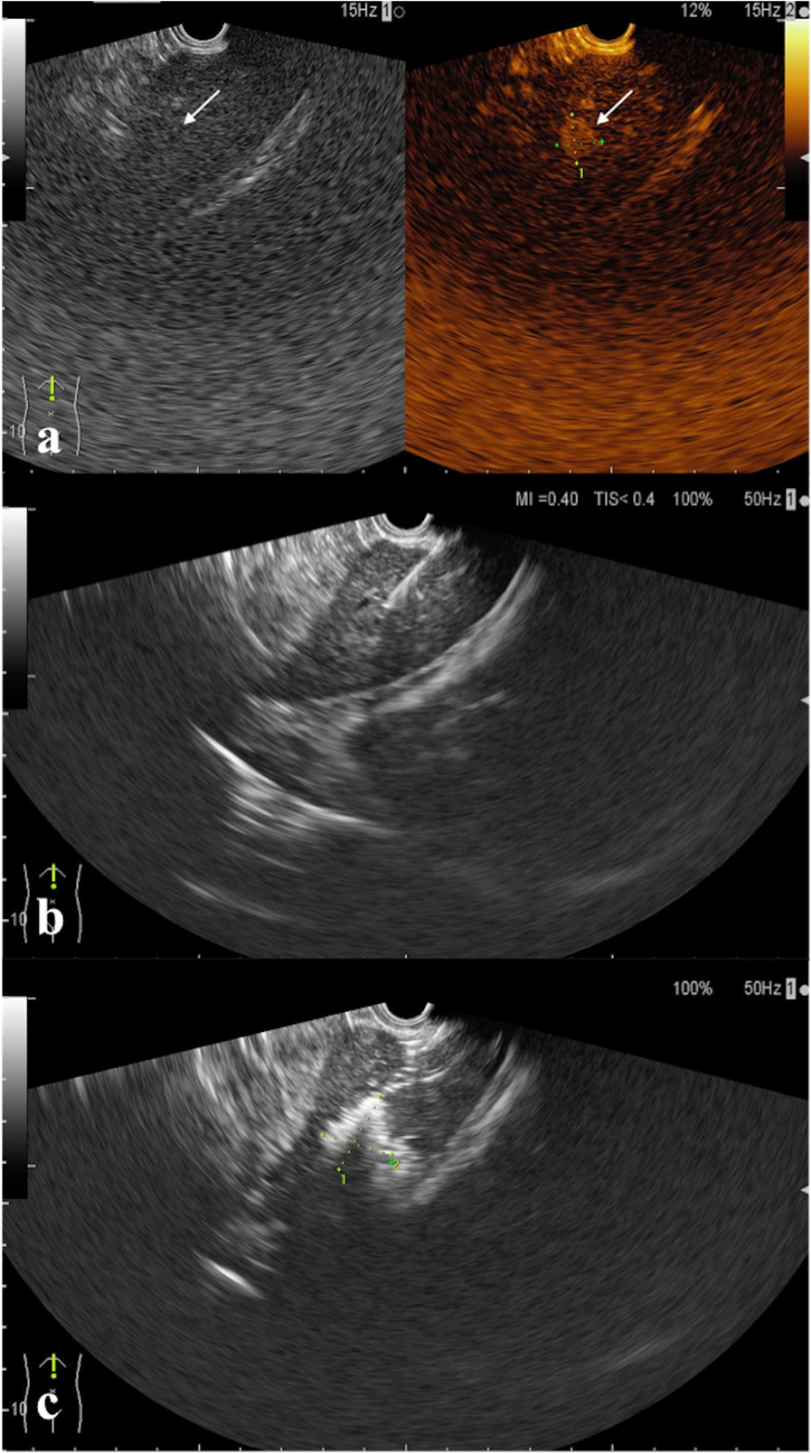
**a** In the arterial phase of contrast-enhanced EUS (26 s), an inconspicuous hypo-echoic lesion sized 1.1 × 1.2 cm is hyper-enhanced (arrowhead). **b** The tip of the laser fibre is clearly visualized and placed at the proximal edge of the target lesion. **c** The treated area is covered by a hyper-echoic zone, the range of which is approximately 2.0 × 1.7 cm in size

**Fig. 3:**
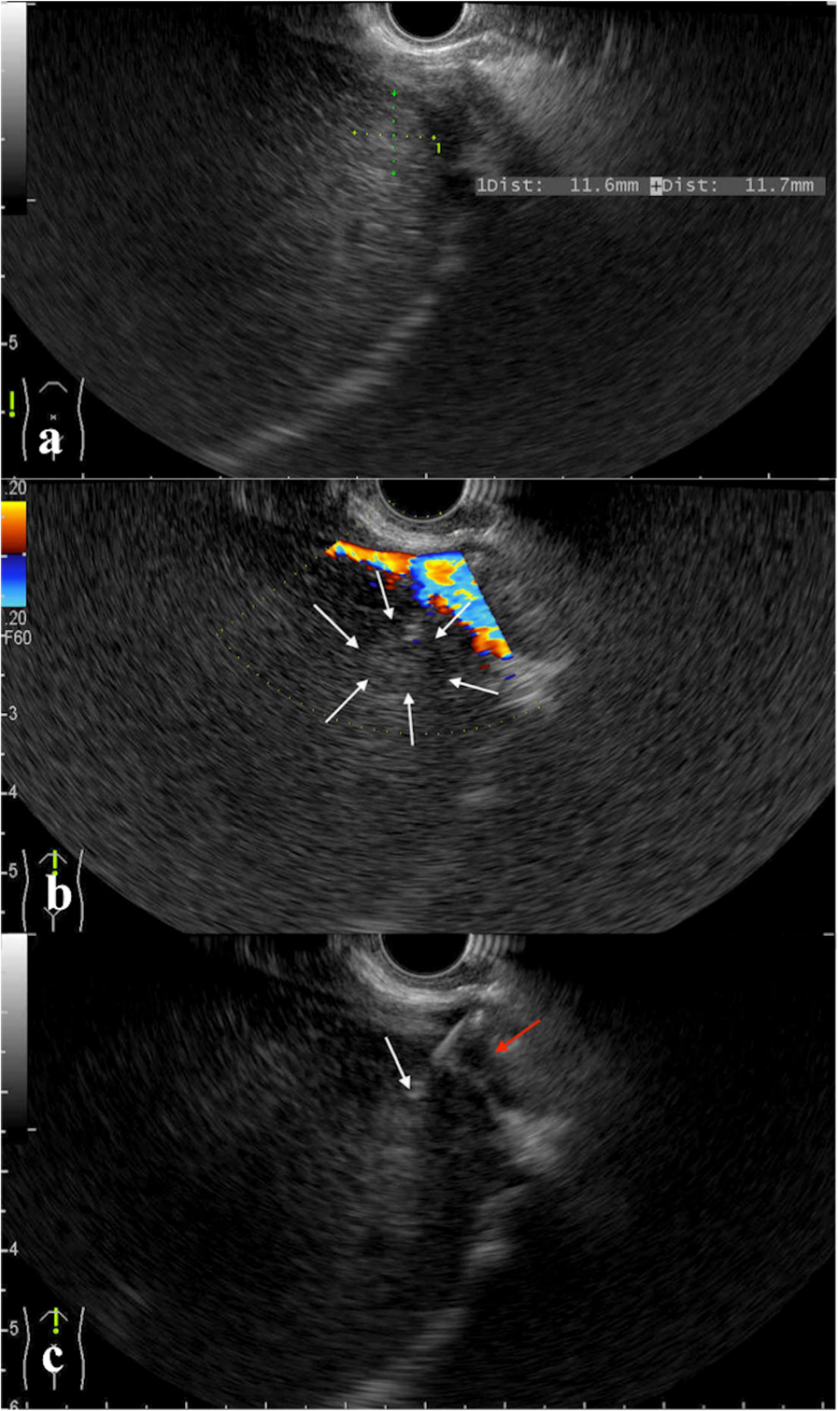
Trans-IVC laser ablation under the guidance of EUS is shown. **a** A HCC lesion located at the hepatic segment VIII is detected using EUS, which was ill-defined in the percutaneous US scanning. **b** The lesion (white arrowheads) is located adjacent to the IVC. **c** A trans-IVC laser puncture is performed for the minimal nature of LA (white arrowhead: the tip of laser fibre, red arrowhead: IVC)

**Fig. 4:**
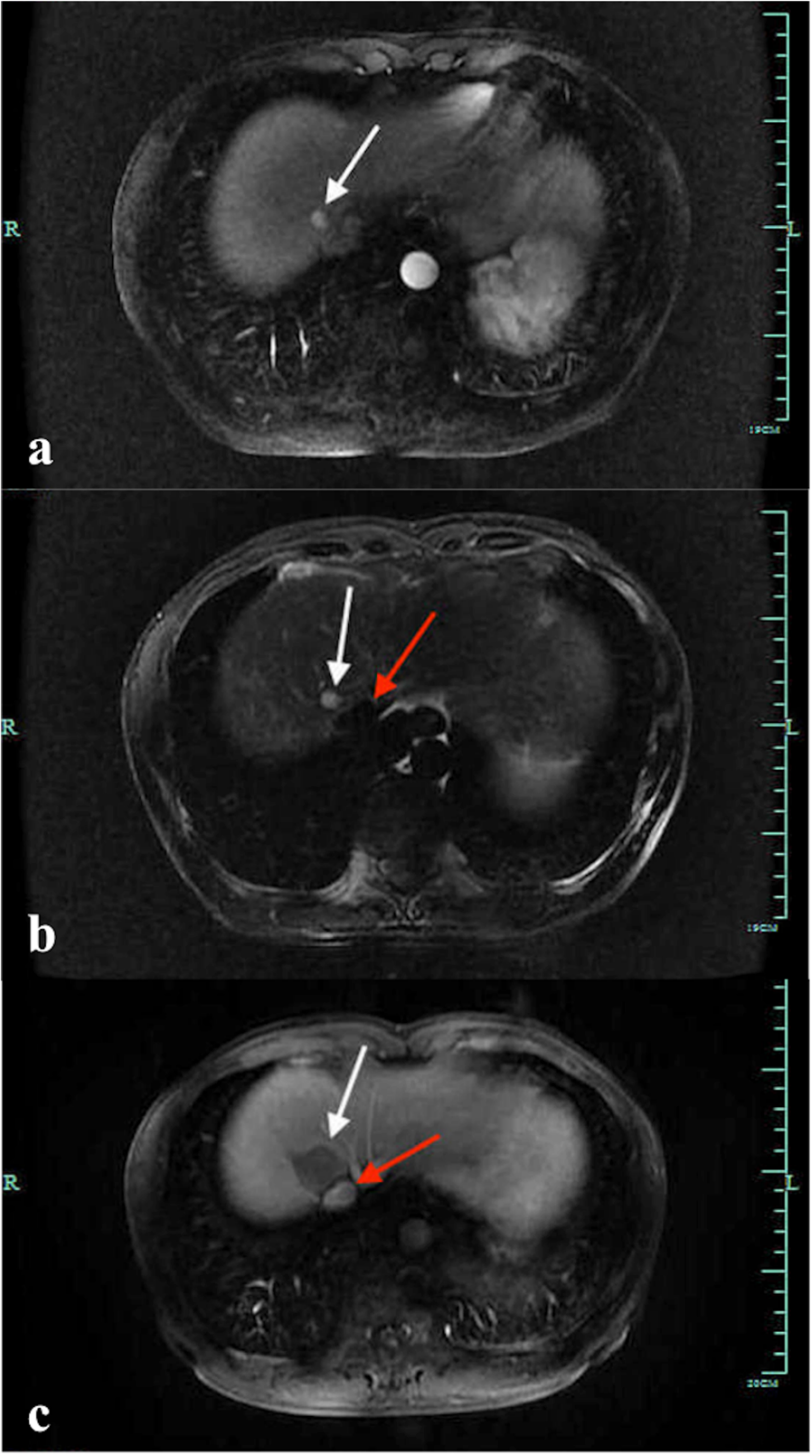
The pre-LA and post-LA MR images of the target lesion are shown. **a** An arterial-phase axial MRI shows a hyper-enhanced lesion was located at the hepatic segment VIII (arrowhead). **b** The lesion (white arrowhead) with a high signal on T2WI is adjacent to the IVC (red arrowhead). **c** A portal-phase axial MRI after trans-IVC LA shows a non-enhanced ablation area (white arrowhead) with intact morphology and blood perfusion of the punctured IVC (red arrowhead)

**Fig. 5:**
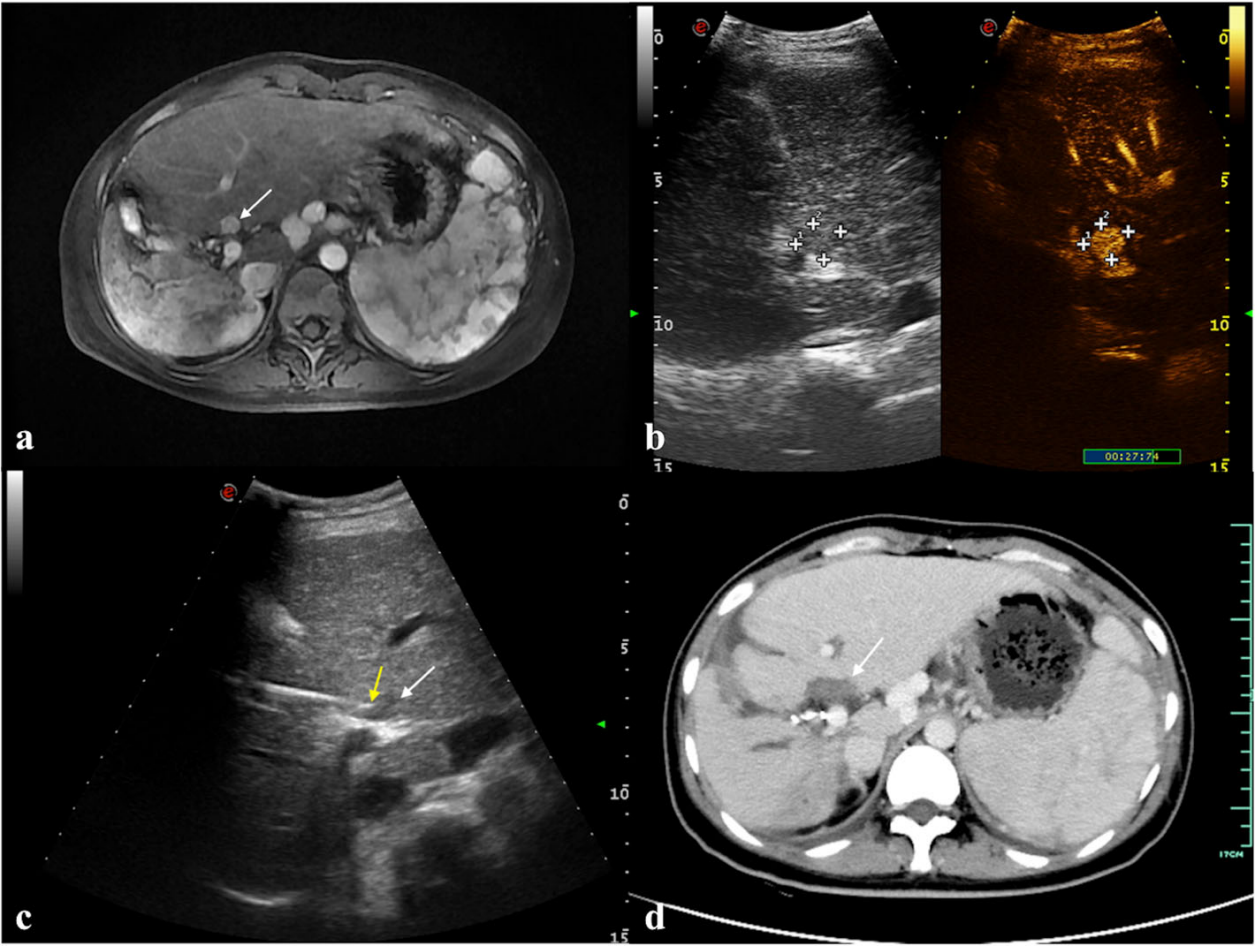
Percutaneous US-guided LA for HCC that is located adjacent to the right branch of the portal vein is shown. **a** An arterial-phase axial MRI shows a subcapsular hyper-enhanced lesion adjacent to the right branch of the portal vein (arrowhead). **b** In the arterial phase of contrast enhanced ultrasonography (CEUS) (27 s), the hypo-echoic lesion is hyper-enhanced with a diameter of 1.5 cm. **c** The tip of the laser fibre (yellow arrowhead) is placed to the proximal edge of the target lesion (white arrowhead). **d** A portal-phase axial CT after US-LA shows that the treated area is non-enhanced. The morphology of the nearby portal vein is intact with normally filled agents (arrowhead)

Among a total of 120 hepatic lesions, 20 (16.7%) lesions were ablated under the guidance of EUS, 69.2% of lesions had a history of thermal ablation, 60.8% had multiple hepatic lesions and 74.2% were diagnosed as HCC. The tumours in the EUS-LA group vs. the US-LA group were more likely to be located in the left lobe of the liver (18, 90.0% vs. 28, 28.0%, respectively, *p* < 0.01). Except for the lesion location, multi-fibre technique (0, 0% vs. 69, 69.0%, *p* < 0.01) and multi-session ablation (4, 20.0% vs. 4, 4.0%, *p* = 0.009) were identified as being significantly different between the EUS-LA and US-LA group, respectively. Figures 1, 2, 3, 4 and 5 show the application of EUS-LA and US-LA to hepatic tumours.

### Treatment response and short-term efficacy

One case of major complication and eight cases of side effects were encountered in the two groups (Table 2). Two cases in the EUS-LA group experienced post-operative nausea and vomiting (PONV). One case in the EUS-LA group and four cases in US-LA group experienced mild-to-moderate pain at the epigastric level. None of these side effects required intensive care, and the condition was remitted in the subsequent few days. A case of subcapsular haemobilia was found 12 h after the US-LA procedure for a lesion adjacent to the hepatic hilum. It presented as progressively decreased blood pressure and was remitted by a timely blood transfusion. The differences in the cumulative incidence of PONV were apparently significant between the two groups (*p* = 0.002); however, a significant difference was not observed in the cumulative incidence of abdominal pain, post-ablation fever or a major complication.

**Table 2:** The complications and side effect occurred in the EUS-LA and US-LA groups

	US-LA (*n* = 100)	EUS-LA (*n* = 20)	*p* value
Side effect	5 (5.0%)	3 (15.0%)	0.102
PONV	0 (0%)	2 (10.0%)	0.001
Abdominal pain	4 (4.0%)	1 (5.0%)	0.838
Post-ablation fever	1 (1.0%)	0 (0%)	1.000
Major complication	1 (1.0%)	0 (0%)	1.000
Sub-capsular haemobilia	1 (1.0%)	0 (0%)	1.000

*PONV* post-operative nausea and vomiting, *EUS* endoscopic ultrasonography, *US* ultrasonography; *LA* laser ablation

All of the 92 patients with 120 hepatic lesions were evaluated for primary endpoint. The CTA was achieved in 94% of patients in the US-LA group and 100% of patients in the EUS-LA group (*p* = 0.261). LTP was investigated in 114 nodules of 87 patients with complete initial ablation; 11 nodules (9.6%) were positive. Five patients (5.4%) were loss to follow-up for 12-month LTP assessment. During the follow-up period, the cumulative incidence of 12-month LTP was observed in 8.5% in the US-LA group and 15.0% in the EUS-LA group with no significant difference between the groups (*p* = 0.372). Except for one patient lost to follow-up, the remaining patients with recurrent lesions were further managed with RFA (*n* = 8), radiotherapy (*n* = 1) and ^125^I implantation (*n =* 1).

Twelve tumour-related and LA-related factors (including guiding methods, history of thermal ablation, multiple hepatic tumours, tumour diameter, tumour diagnosis, poorly differentiated tumour, recurrent tumour, tumour at a high-risk position, tumour in the left lobe, multi-fibre technique, multi-session ablation and delivered energy) for LTP were evaluated using multivariate analysis. The logistic regression model identified that the tumour diagnosis (*p* = 0.004; 95% CI, 0.039–0.547) was the only independent risk factor associated with post-LA LTP. The hazard ratio of LTP was 1.9 times higher for patients with cholangiocarcinoma or metastatic tumours compared to those with HCC.

## Discussion

To the extent of our knowledge, this is the first population series to evaluate the clinical application of percutaneous US- and EUS-guided LA for unresectable hepatic tumours and compare the differences in the incidences of complications, CTA and 12-month LTP. Our study showed that similar treatment response and short-term efficacy were observed in the US-LA and EUS-LA groups.

RFA currently represents the optimal choice for the early stage of HCC or unresectable hepatic tumours that affords better local tumour control and clinical outcomes. LA is the least investigated but the most promising thermo-based technique by far according to its minimal nature and efficient light penetration. The introduction of new therapeutic EUS techniques such as EUS-guided injection or LA is still under development, and it is only used by some specialized experts or centres.

For the limitation of the scanning range and angle of EUS, the ablated tumours in the EUS group were more likely to be located in the left lobe of the liver than those in percutaneous US group. In our practice, the percutaneous puncture method from the subxiphoid or subcostal point to the left lobe of the liver was sometimes more susceptible to respiration-accompanied liver movement, which increased the procedural difficulty and risk. In accordance with the result of Grasso’s study in 2010, such lesions, especially in the caudate lobe or left external lobe close to the stomach, could be accessed via EUS-guided LA [6]. When the tumours are located in poorly accessible regions of the liver to which an appropriate puncture point or optimal needle placement from the percutaneous approach is lacking, a second try using an endoscopic approach should be considered.

The multi-fibre LA technique was only applicable via the percutaneous approach. The needles could be inserted in a parallel fashion, facilitating the distance to achieve the maximum configurations. A needle guidance device for the US transducer is commercially available. It can be fixed on the transducer and has separate channels for needle insertion in a prefixed fashion and needle distance, making the fine-needle insertion easier and quicker [17]. According to our experience, in situations where the tumour is larger than 2.0 cm or has an irregular shape, the multi-fibre technique was usually adopted to avoid a repeated puncture intra-operatively or needle adjustment. In the EUS-guided procedure, the single-fibre technique with multi-session LA was used instead of the multi-fibre technique, so that the applied fibres and ablation sessions were shown to be significantly different between the two groups. A precise ablative design appeared to be more of a limiting issue for achieving CTA.

LTP, one of the best criteria for the measurement of the efficacy of thermoablation, was reported to be increased if the tumour was > 3.0 cm or the tumour-free margin was not sufficient wherever it was located. In a recent study, the recommended laser ablative margin in patients with HCC > 4 cm was increased to at least 7.5 mm [18–21]. Compared to RFA, the rate of CTA, deemed as the only factor associated with improved survival, was equal to the application of LA in cases of small HCC [3]. Di et al. reported that the LTP to HCC was 25.7% in RFA patients and 22.9% in LA patients and the mean time to LTP was 42 months in RFA and 46.7 months in LA [4]. At the overall median follow-up of 81 months, the LTP to completely ablated HCC nodules was 10.6% that was free from the possible influence of a high-risk tumour location [19]. By a new guidance system, the LTP to LA was observed in 16.1% at a mean LTP time of 26 months [17]. The tumour size was demonstrated as the main predictor for LTP after percutaneous thermal ablation of the liver tumours, especially for the tumour larger than 3 cm [18, 20, 21]. In our series, the hepatic tumours were classified as HCC and non-HCC nodules, and the LA for non-HCC nodules was proved to be the only risk factor for the incidence of 12-month LTP. However, the mean diameter of tumours in both the US-LA and EUS-LA groups was smaller than 2 cm, thus the diameter was not suggested.

With the development of EUS-guided FNA, an increased number of studies have focused on therapeutic EUS. Reported cases of EUS-guided ^125^I seed brachytherapy, ethanol injection and LA have revealed that in the treatment of those hard-to-reach lesions using a conventional approach, EUS appeared as an endoscopic modality for the purpose of monitoring and guiding the procedure. These newly attempted EUS-guided fine-needle interventions were feasible and effective. The promising local tumour control was comparable to that achieved via percutaneous US-guided LA [7, 12]. CEUS was also helpful during EUS-guided LA procedure to assess the ablation margin, identify the remaining vital tumour and monitor the perfusion of adjacent major vessels.

The US- and EUS-accompanied morbidities in our study (one case of a major complication, subcapsular haemobilia, 0.8%) were acceptable and lower than those in the recently published studies. Previously, reported major complications occurred in 1.5% of LA sessions, which was associated with excess energy, high-risk location and abnormal prothrombin time [15]. Some specific major complications that accompanied EUS-guided LA were gastrointestinal perforation and bleeding; however, in our study, no major complications and procedure-related deaths occurred in the EUS-LA group [13]. It is noteworthy that in the procedure of LA of the tumours located in the hepatic hilum, the heart rate and blood pressure should be cautiously monitored for the possible accompanied vasovagal reaction.

The study has several limitations. First, this is not a multi-centre design study, and the sample size of the included patients was relatively small. Second, the visualization of EUS was limited in certain segments of the liver, and EUS-LA could only be applied in the lesions within the field of EUS scanning. Therefore, the included cases could not be randomly assigned into the two therapeutic arms. The comparison bias leading by the heterogeneity between the two arms was hardly to be avoided. Finally, the therapeutic strategies and operations were determined by two operators, which may lead to selective bias. Further studies addressing these limitations are necessary.

## Conclusions

The EUS-LA group had a similar treatment response and short-term efficacy to those of the US-LA group. For the limitation of the scanning range and angle of EUS, the single-fibre technique with multi-session LA was applicable to hepatic lesions. A hybrid LA approach to nonsurgical hepatic tumours appeared to be reasonable.

## Data Availability

The datasets used or analyzed during the current study are available from the corresponding author on reasonable request.

## Abbreviations

CT: Computed tomography
CTA: Complete tumour ablation
EUS: Endoscopic ultrasonography
FNA: Fine-needle aspiration
FNI: Fine-needle injection
HCC: Hepatocellular carcinoma
ICC: Intrahepatic cholangiocarcinoma
IVC: Inferior vena cava
LA: Laser ablation
LTP: Local tumour progression
MRI: Magnetic resonance imaging
Nd:YAG: Neodymium: Yttrium Aluminium Garnet
PONV: Post-operative nausea and vomiting
RFA: Radiofrequency ablation
US: Ultrasonography
